# Role of Methylation in *Period2* (*PER2*) Transcription in the Context of the Presence or Absence of Light Signals: Natural and Chemical—Studies on the Pig Model

**DOI:** 10.3390/ijms22157796

**Published:** 2021-07-21

**Authors:** Przemysław Gilun, Krzysztof Flisikowski, Tatiana Flisikowska, Joanna Kwiatkowska, Barbara Wąsowska, Magdalena Koziorowska-Gilun

**Affiliations:** 1 Department of Local Physiological Regulations, Institute of Animal Reproduction and Food Research, Polish Academy of Sciences, 10-748 Olsztyn, Poland; b.wasowska@pan.olsztyn.pl; 2 School of Life Sciences, Chair of Livestock Biotechnology, Technical University of Munich, D-85354 Freising, Germany; flisikowski@wzw.tum.de (K.F.); tatiana.flisikowska@wzw.tum.de (T.F.); 3 Department of Neurosurgery, School of Medicine, University of Warmia and Mazury, 10-719 Olsztyn, Poland; asia.kwiatkowska87@gmail.com; 4 Department of Animal Biochemistry and Biotechnology, Faculty of Animal Bioengineering, University of Warmia and Mazury, 10-719 Olsztyn, Poland; magda.koziorowska@uwm.edu.pl

**Keywords:** gene expression, E-box, methylation, *PER2*, carbon monoxide, oscillation

## Abstract

It has been proposed that carbon monoxide (CO) is a chemical light carrier that is transferred by the humoral pathway from the retina to the brain. Here, we aimed to study how deeply CO is involved in regulating the expression of *Period2* gene (*PER2*), one of the genes maintaining the intrinsic biological clock. In our in vivo experiment, we studied whether CO may be a chemical signal and is also equivalent to natural light in three groups of pigs: Normal: housed in natural conditions without any procedures, Control: adapted and kept in constant darkness, infused with blank plasma, and CO treated: adapted and kept in constant darkness infused with CO-enriched plasma. After the experiment, the animals were slaughtered at two times of day: 12 p.m. and 12 a.m. Next, hypothalamus samples were collected. Quantitative PCR, the DNA methylation of the promoter sequence containing enhancers (E-box) and a functional analysis of the *PER2* promoter was performed. qPCR showed a differential pattern of *PER2* mRNA expression at daytime oscillation in the examined groups. Pyrosequencing revealed daytime changes in the methylation level of regulatory sites of the examined sequence. Luciferase reporter assay confirmed that E-boxes (CANNTG) drive the expression of the porcine *PER2* in vitro. In conclusion, changes in methylation over 24 h may regulate the oscillatory manner of *PER2* expression.

## 1. Introduction

Many physiological processes are cyclic and depend directly or indirectly on the intrinsic biological clock. The main biological clock in mammals is located in suprachiasmatic nuclei (SCN) in the pre-optic part of the hypothalamus and is modulated endogenously by clock-related genes such as *PER1*, *PER2*, *PER3*, *CRY1* and *CRY2*. These genes are regulated by a heterodimer of the specific transcription factors BMAL1/CLOCK that react with E-box enhancers in the promoter region of genes (*PER1–3* and *CRY1–2*), creating a core loop. The PER and CRY proteins in turn heterodimerize and translocate to the nucleus to inhibit BMAL1/CLOCK activity. This is a cell-autonomous molecular oscillator containing a transcription-translational feedback loop (TTFL) that displays a roughly 24 h cycle. Circadian rhythm depends on external cues called zeitgebers, and the most important is light [1,2,3]. Sunlight falling on the retina starts the process of neural light transmission to the hypothalamus to SCN [4]. It has been reported that the retina can simultaneously produce carbon monoxide (CO) as a chemical light carrier and that this production depends on light intensity [5,6,7]. Further CO is transferred via the humoral pathway to the hypothalamus and modulates the biological clock as a „chemical light signal” independently from a neural light signal [8].

The *Period2* gene is a member of the Period family (*PER1–3*). These genes cooperate with cryptochromes in TTFL in SCN to form the main biological clock. *PER2* is also expressed in peripheral tissues, creating peripheral clocks that, in turn, depend on the main biological clock [9]. *PER2* plays a more dominant role than *PER1* in the circadian rhythm that affects the central and peripheral nervous systems [10,11]. Furthermore, *Per2*/PER2 is involved in many such processes in the nervous system, such as neurotransmitter regulation (GABA, dopamine, glutamate and serotonin) and prolactin production, as well as sleep and food anticipation. Some authors indicate that *PER2* plays a role in neurodegenerative diseases, drug addiction and depression [11,12]. *PER2* is regulated through the promoter sequences containing enhancer E-boxes [CANNTG] [13] and D-box (daytime expression enhancer) and also CRE (cAMP-response element) [14,15] in gene sequences downstream from ATG (start of translation).

Functional clock gene proteins such as BMAL1, CLOCK and NPAS2, as well as PER2, REV-ERB α and β belong to a group of proteins called basic helix-loop-helix PER-ARNT-SIM (bHLH-PAS), containing PAS A and PAS B domains that form functional protein dimers with different biological functions. These domains are able to bind heme groups with Fe(II)-valent iron, which acts as a cofactor and influences the function of these proteins. Binding heme groups to Fe(II) at the active center sensitizes these proteins to CO, which, by binding to heme, exerts such biological effects as inhibiting the dimerization of BMAL1:NPAS2 proteins [16,17,18].

The aim of this research was to study the regulation of *PER2* expression over a 12 h period. We used pigs for the experiment, because they have a similar circadian rhythm as humans, in which daylight activity and housing with artificial light influence the biological clock. The pig brain is big enough for us to obtain an appropriate amount of samples from the hypothalamic preoptic area with SCN but without other nuclei; i.e., paraventricular (PVN in the dorsal part of the hypothalamus) or arcuate (ARC in the ventral part of the hypothalamus). We used CO as a chemical light signal because our recent research showed that the CO level in blood depends on time and light intensity and that it can modulate the biological clock in the hypothalamus [5,8]. Our experimental model assumed that only a chemical light signal or lack of it will be a cue for the organism to synchronize the main biological clock, which is why we used animals adapted to constant darkness.

We hypothesized that carbon monoxide delivered by the humoral pathway to the hypothalamus will change the *PER2* promoter methylation in order to organize the mechanistic function of the main biological clock. 

## 2. Results

### 2.1. PER2 Gene Expression

**Figure 1:**
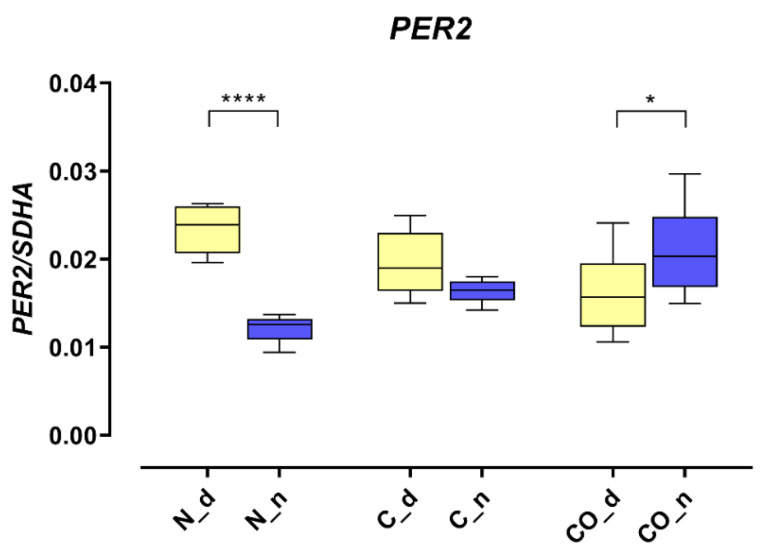
The *PER2* mRNA expression in the preoptic part of the hypothalamus that contained suprachiasmatic nuclei in the normal (N), control (C) and CO-treated groups. The data were normalized by dividing the expression of the target gene by average expression of the housekeeping gene *SDHA* (succinate dehydrogenase). The bars are represented as mean ± SEM in arbitrary units. The *PER2* mRNA expression obtained in the tissues of the hypothalamus during the day (yellow whisker boxes) and night (blue whisker boxes) were compared in particular studies groups. The asterisks indicate significant differences as follows: *—*p* < 0.05; ****—*p* < 0.0001.

The *PER2* mRNA expression in the hypothalamus was 2.5-fold higher (*p* < 0.0001) during the day in the normal group; this kind of pattern of daytime expression is called oscillation. In the control group, we did not observe statistically significant changes between day and night, and oscillation was damped. The *PER2* expression trend reversed in the CO-treated group; during the night, it was significantly higher (*p* < 0.05) compared to expression during the day, and oscillation reversed at night (Figure 1).

### 2.2. Methylation Level

**Figure 2:**
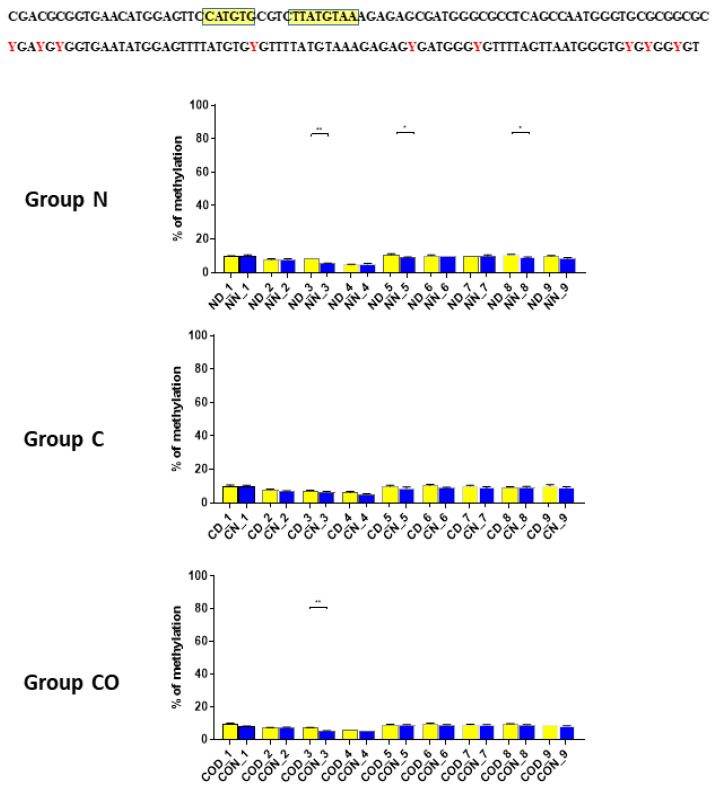
The difference between day (yellow bar) and night (blue bar) methylation levels in the hypothalamus in the normal (N), control (C) and CO-treated groups. The region closest to the ATG (−1–−79 bp) canonical E-box was hypomethylated. In this region, the difference between methylation was observed in the normal group at the 3rd, 5th and 8th CpG positions. The CO-treated group showed a significant change in the methylation level in the 3rd CpG position. The bars are represented as mean ± SEM. The asterisks indicate significant differences as follows: *—*p* < 0.05; **—*p* < 0.01. At the top of the figure is the examined sequence: original (**top**) with yellow highlighted sequences E-box and D-box and bisulfite converted with highlighted methylation position (**bottom**).

**Figure 3:**
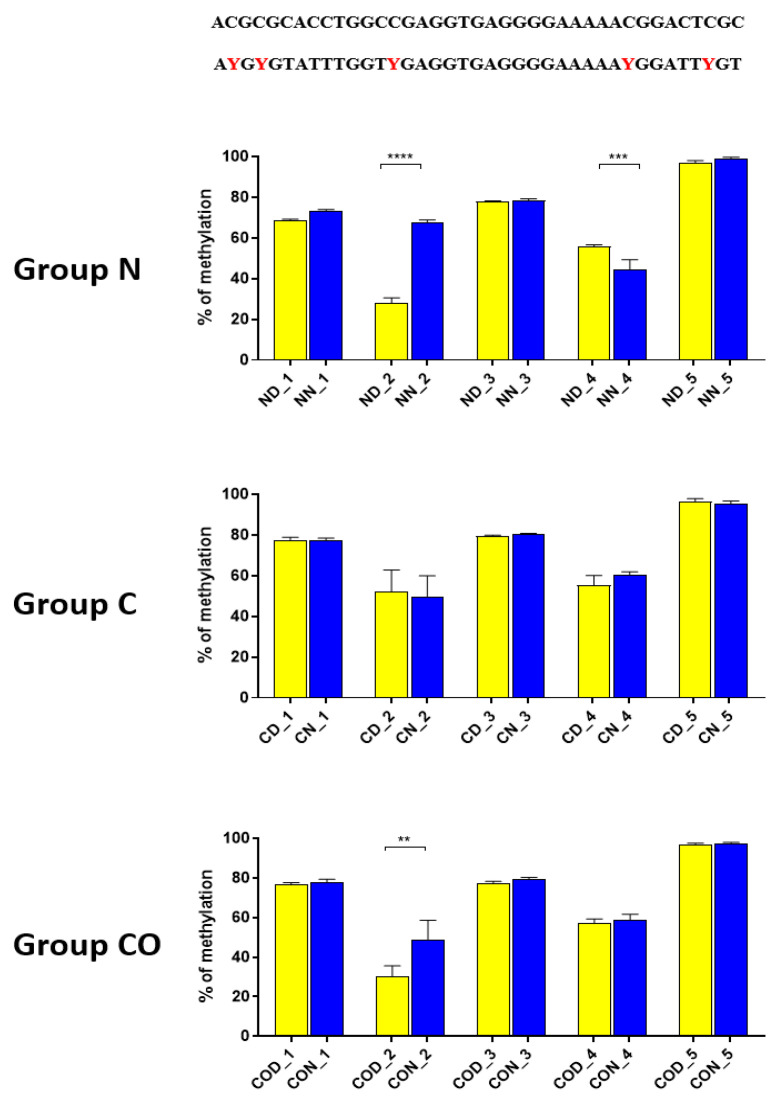
The difference between day (yellow bar) and night (blue bar) methylation levels in the hypothalamus in the normal (N), control (C) and CO-treated groups. The highest methylation level was observed in −1185–−1224 bp, where it was on 80–100%. The methylation level was significantly higher at the 2nd and 4th CpG position in hypothalamic DNA samples collected at night in the normal and in 2nd CpG position in the CO-treated group. The bars are represented as mean ± SEM. The asterisks indicate significant differences as follows: **—*p* < 0.01; ***—*p* < 0.001; ****—*p* < 0.0001. At the top of the figure is the examined sequence: original (**top**) and bisulfite converted with highlighted methylation position (**bottom**).

**Figure 4:**
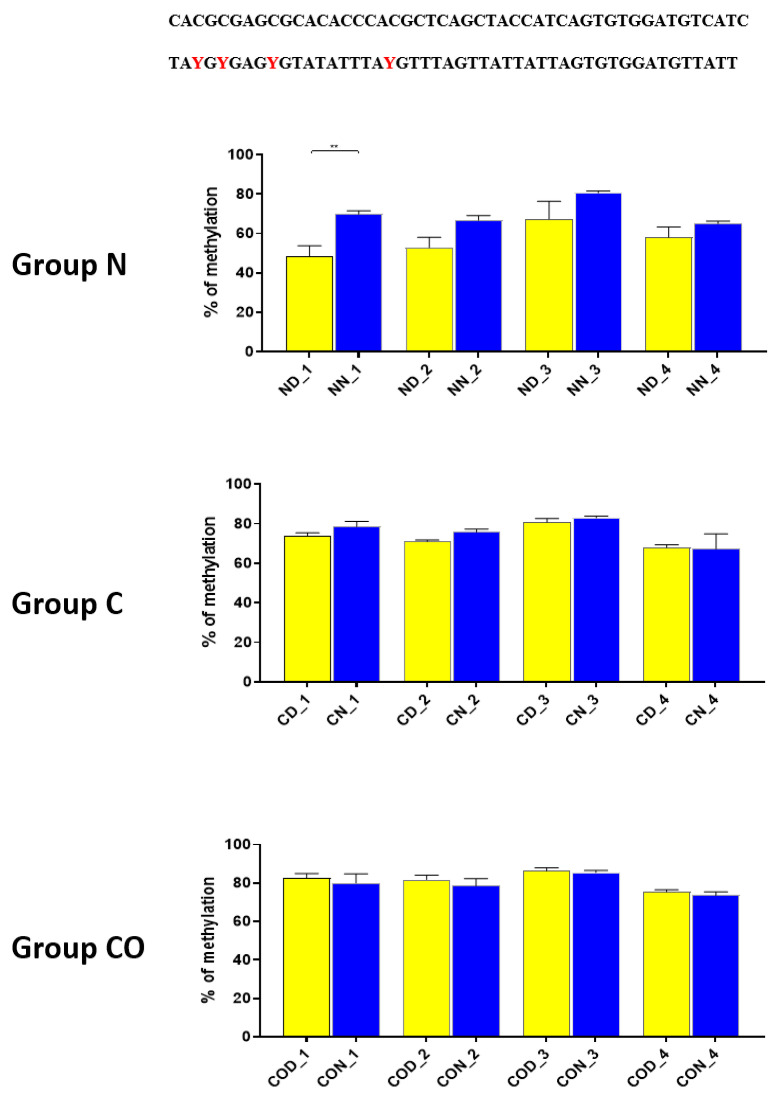
The difference between day (yellow bar) and night (blue bar) methylation levels in the hypothalamus in the normal (N), control (C) and CO-treated groups. The −2000—−2054 bp region methylation level was high at 60–80% and varied significantly between day and night only in the normal group in position 1 of CpG. The bars are represented as mean ± SEM. The asterisks indicate significant differences: **—*p* < 0.01. At the top of the figure is the examined sequence: original (**top**) and bisulfite converted with highlighted methylation position (**bottom**).

**Figure 5:**
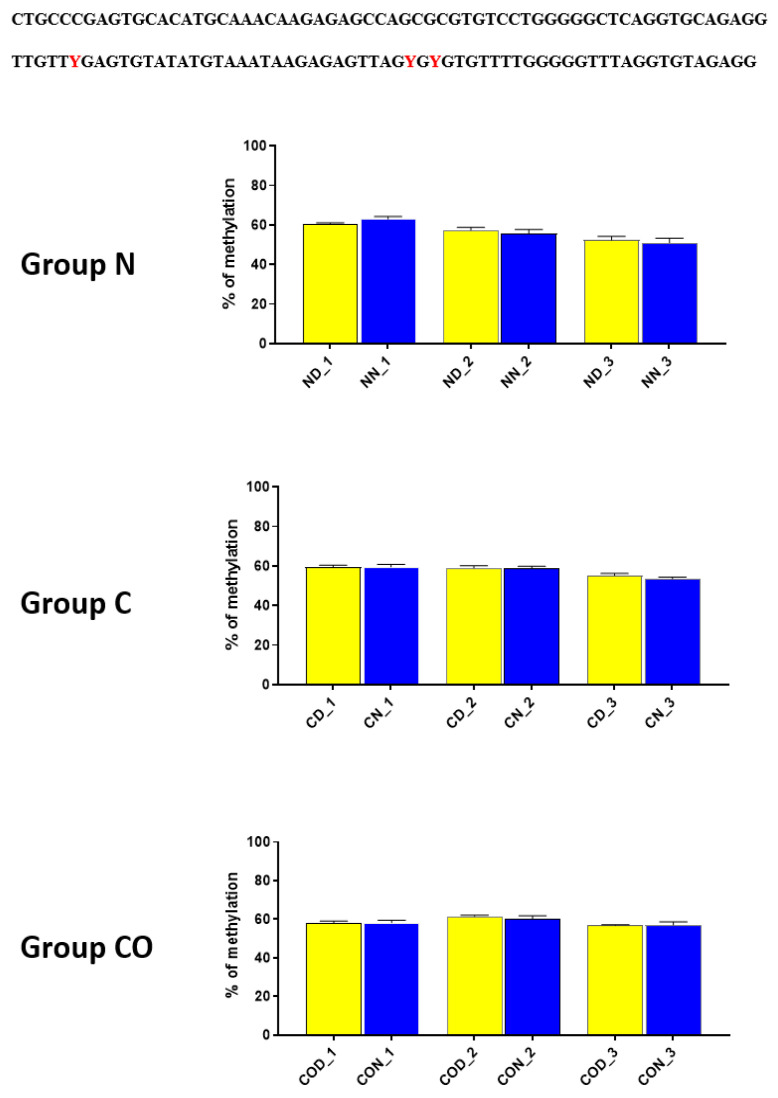
The difference between day (yellow bar) and night (blue bar) methylation levels in the normal (N), control (C) and CO-treated groups of the hypothalamus. The methylation level between −2402–−2464 bp in all the experimental groups was approximately 60%. There were no differences in methylation levels between day and night in this region. The bars are represented as mean ± SEM. At the top of the figure is the examined sequence: original (**top**) and bisulfite converted with highlighted methylation position (**bottom**).

Four different promotor sequence regions were studied: −1–−79 bp, −1185–−1224 bp, −2000–−2054 bp and −2402–−2464 bp. The region closest to the ATG (−1–−79 bp) canonical E-box was hypomethylated. The DNA methylation was around 10%. In this region, the difference between night and day methylation levels was observed in the normal group at the 3rd (*p* < 0.01), 5th (*p* < 0.05) and 8th (*p* < 0.05) CpG positions. The CO-treated group showed a change in the methylation level at the 3rd CpG position. All the described hypothalamus samples exhibited a higher methylation level during the day. In the control group, the methylation level did not differ at any of the CpG positions (Figure 2). The highest methylation level (80–100%) was observed between −1185–−1224 bp. The methylation level was significantly higher at the 2nd CpG position in hypothalamic DNA samples collected at night in the normal (*p* < 0.001) and CO-treated groups (*p* < 0.01). The methylation level in the normal group at the 4th CpG position was also higher at night (*p* < 0.01) (Figure 3). The −2000–−2054 bp region methylation level was high at 60–80% and varied significantly between day and night only in the normal group at the 1st CpG position (*p* < 0.001) (Figure 4). The methylation level between −2402–−2464 bp in all the experimental groups was approximately 60%. There were no differences in methylation between day and night in this region (Figure 5). 

**Figure 6:**
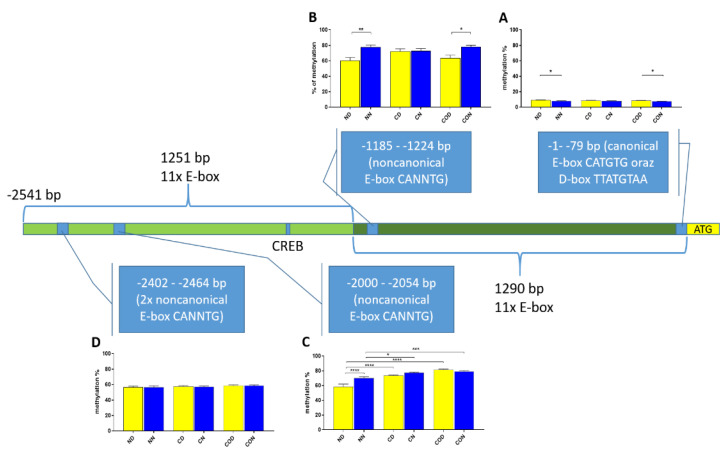
Scheme of promoter sequence −2500 bp downstream ATG with changes in daily total methylation level of the examined sites, −1–−79 bp (**A**), −1185–−1224 bp (**B**), −2000–−2054 bp (**C**), −2402–−2464 bp, (**D**) in the normal (N), control (C) and CO-treated groups. The differences in total methylation level were compared in each group between day (yellow bar) and night (blue bar). The bars are represented as mean ± SEM. The asterisks indicate significant differences as follows: *—*p* < 0.05; **—*p* < 0.01; ***—*p* < 0.001; ****—*p* < 0.0001.

We also analyzed the overall methylation level in each of the four regions (Figure 6) and observed that daily oscillation in region −1–−79 bp differed statistically (*p* < 0.05) between day and night in normal and CO-treated groups. In the control group, neither daily oscillation nor differences between groups were observed (Figure 6A). We observed daily oscillation in region −1185–−1224 bp in the normal group (*p* < 0.01) and the CO-treated group (*p* < 0.05) but not in the control group (Figure 6B). In the third examined region, −2000–−2054 bp, we observed the oscillation of the total methylation level only in the normal group (*p* > 0.0001). We observed an increased methylation level in the day in the control and CO-treated groups (*p* < 0.0001) for both groups compared to the normal group, while, at night, we observed increased levels in the control group (*p* < 0.05) and CO-treated groups (*p* < 0.001) compared to the normal group (Figure 6C). In the last examined region −2402–−2464 bp, we did not observe any differences (Figure 6D).

### 2.3. Functional Analysis

**Figure 7:**
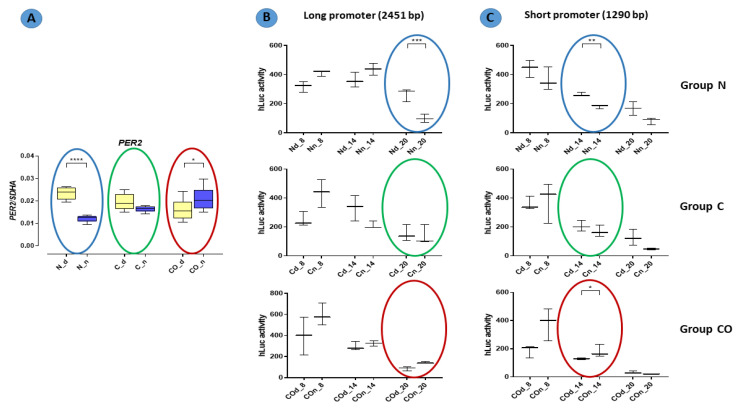
Promoter activity in the normal (N), control (C) and CO-treated groups. The significant differences between day (Nd, Cd, COd) and night (Nn, Cn, COn) were analyzed in each studied group. In functional assay with the HEK 293 cell line, promoter activity was measured with relative luminescence values in 6 h intervals (8 a.m., 2 p.m., 8 p.m.). Oscillation in promoter activity was observed in both the long (**B**) and short (**C**) promoter sequences. Colored circles mark the time points corresponding to the pattern expression obtained in qPCR (**A**) (repeated Figure 1). The asterisks indicate significant differences as follows: *—*p* < 0.05; **—*p* < 0.01; ***—*p* < 0.001; ****—*p* < 0.0001.

In functional assay with the HEK 293 cell line, promoter activity was measured with relative luminescence values in 6 h intervals. Oscillation in promoter activity was observed in both the long and short promoter sequences (Figure 7). There were differences between the expression levels in the promoter sequence from DNA obtained from tissue isolated at night and day. The activity of the promoter in tissues isolated at night had lower expression level in the normal group at 8:00 PM with a long sequence (*p* < 0.001) and at 2:00 PM with a short sequence. Moreover, at 2:00 p.m., the short promoter showed a higher activity in samples isolated at night in the CO-treated group (*p* < 0.05).

## 3. Discussion

The presented data obtained from the experimental pigs showed that *PER2* mRNA changed the pattern of its own expression in the hypothalamic preoptic area over a 24 h period (Figure 1). Animals from group N (normal) displayed a valid pattern of *PER2* gene-expression oscillation in mRNA; however, the animals from group C (control) and CO (CO-treated) showed notable changes. The level of mRNA expression did not change significantly, but we observed a lack of oscillation in group C and reverse oscillation in group CO. This situation is similar to data previously published by Gilun et al. [8], in which a lack of the main environmental cue, which was light, caused the curve of oscillation in group C to flatten. The addition of CO to infused plasma as a chemical light signal, i.e., light dependent in natural light condition, caused *PER2* mRNA oscillation to be reinstated but in a reverse manner. The phase inversion in the animals in group CO might be explained by the fact that the *PER2* oscillation cycle was faster than 24 h, which would explain why, after 72 h of continuous infusion, the cycles would be consecutively shorter. In this case, the hypothalamic SCN received a neural signal concerning the lack of light and a chemical light signal transferred with blood. In physiological conditions the retina transmits to the brain a neural light signal; called a non-forming image [19] and a chemical signal through the humoral pathway, where the retina releases more carbon monoxide when exposed to light [5,6,7]. In natural conditions, the daytime increase in CO modulates the main biological clock [8]. The oscillatory pattern of *PER2* expression is necessary because *PER2* regulates the transcription of Rev-erbα and Bmal1 and is associated with the degradation of the CLOCK-BMAL1 heterodimer. Because the mechanistic functioning of the circadian rhythm depends on two interlocked loops, 24 h oscillation of clock genes is essential to creating this machinery [20,21,22]. CO as a cofactor regulates the dimerization process of such heme-dependent TFs as BMAL1, CLOCK and NPAS2. The bioavailability of CO might be a very efficiently modulator of PER2 gene expression but only in the state when two TF proteins are not dimerized together [16]. However, we know the states when the TFs are anchored to the promoter, and CO probably cannot prevent this function through binding to heme, but the other clock protein (CRY) can dissociate TF’s complex from the promoter. Even though CRY can interrupt TF’s function and the exposure of TF’s complex to CO, PER protein sufficiently prevents this situation through the dissociate CRY from TF’s dimer anchored to the promoter [23]. The timing of the transcription of *PER2* mRNA expression oscillation is regulated by the E-box and D-box structures. The circadian rhythm of *PER2* depends on the number of functional E-boxes in the promoter sequence [24].

After we analyzed the experimental data for *PER2* mRNA expression, we checked whether the 12 h variable of this gene could be regulated epigenetically. The obtained data revealed that methylation in each selected region was different and changed daily as well as among experimental groups. The DNA sequence placed near ATG was methylated only about 10%, and we observed that daily oscillation in methylation level in the normal and CO-treated groups was statistically significant (Figure 2). This phenomenon was associated with both neural and humoral light signals. 

In group C, we did not observe any oscillation due to a lack of light signal in this group. A closer analysis of 9 CpG sites in this sequence showed a daily oscillation in position 3 in the normal and CO-treated groups and in positions 5 and 8 only in the normal group. Between positions 3 and 5 we identified E-box [CATGTG] [24,25] and D-box [TTATGTAA] [14,26,27]. CpG located between E-box and D-box (position 4) was the least methylated of all nine examined positions. The presence of a generally low methylation level in this region of the promoter suggests its regulatory function. Additionally, the oscillatory methylation effect at both ends of the functional sequence showed us that environmental cues modulate gene expression through the epigenetic pathways. This is because the methylation of CpG sites was effective and reduced binding affinity to heterodimeric proteins [28]. However, in our case, CpG positions, methylated in a 12 h period, were located on a flank of the examined regulatory sequence and might have played only a co-regulatory function (Figure 2). 

The question arises why this site is not sufficiently recognized by DNA methyl transferase 3 a/b (Dnmt3a/b) despite the many CpG islands in this sequence. We know that the predominant role of Dnmt3a/b is de novo methylation, and its interaction with transcription factors is required for targeted DNA methylation [29,30]. The c-MYC transcription factor interests us because this bHLH (basic Helix-loop-Helix) protein recognizes the E-box sequence (CASSTG) and its main function is cell proliferation and differentiation [29,31,32]. In our experiment, in the highly methylated sequence from −1185 to −2464 bp downstream ATG, we identified eleven sites containing the CASSTG sequence, which are potential targets for DNMT3a/MYC heterodimer. 

Even though we do not have any ChIP data of this sequence, we assume that so many E-boxes in this part of the promoter means that c-MYC may compete with the CLOCK/BMAL1 heterodimer and affect the circadian regulation of *PER2* through overlapping. Moreover, the canonical E-box (CATGTG) placed in the low methylated sequence (−1 to −79 bp downstream ATG) is not preferred by MYC [25], which explains why this sequence is not “protected” throughout the methylation against MYC despite high amounts of CpGs in that short fragment. Hervouet et al. [29] showed that MYC is not the only protein that cooperates with DNMT3a in targeted DNA methylation and described sixty-eight interacting proteins, including CREB (cAMP-response element (CRE)—binding) and ATF4 (activating transcription factor 4). These two proteins were of our particular interest because we also identified a sequence recognized by CREB (TGACGTCA), which is located from −1478 to −1486 bp downstream of ATG, between two highly methylated fragments (Figure 6). Myc E-boxes are also located on both sides of this CREB sequence. However, the direct perimeter of this sequence has only two CpGs, of which one is one of the two identified positions inside the CREB sequence, which means that the particular CpG position can be methylated by 12-h variable.

We assumed this, because in both upstream and downstream of CREB sequence, we discovered that the total methylation level (for each examined fragment, Figure 6) can oscillate and that there are sustained changes between the day and night methylation levels, especially in group N and group CO. Koyanagi et al. [15] revealed that ATF4 is bound to the CRE sequence in the 5′-flanking region of the *PER2* gene and sustains rhythmic expression of the *PER2* gene along with constitutively bound CLOCK in E-box. ATF4 also acts as an output signal for regulating the oscillation of the CRE-mediated signal, which only occurs together with CLOCK-mediated gene expression. Without CLOCK, the rhythmicity of *PER2* expression is strongly reduced.

Despite the knowledge that DNMT3a with MYC/CREB/ATF4 is required for targeted methylation and that sustained rhythmical *PER2* expression is co-regulated through the ATF4, we discovered the phenomenon of daily changes of methylation in a sequence recognized by DNMT3a/MYC. We suggest that the availability of a CREB sequence for CREB/ATF4 is also regulated by the daily methylation changes of the CpG position inside the CRE sequence through either the DNMT3a/ATF4 or DNMT3a/CREB. This kind of regulation explains why CLOCK is indispensable in rhythmic *PER2* expression, and our data on changes in methylation levels supports this concept.

A detailed analysis of each assayed CpG positions in the highly methylated fragments revealed that 12 h changes occurred in positions 2 (Figure 3) and 1 (Figure 4) in methylation level dependent on light availability. An analysis of these positions showed that it concerns a specific sequence in both (CACGCG) sites when one CG is methylated by a 12 h variable. This is an N-box sequence and is recognized by the *HES1* (hairy and enhancer split 1) protein that belongs to the bHLH transcription factor [29]. This protein regulates and suppresses other bHLH neuronal factors in developmental and differentiation processes [32]. Moreover these processes are regulated by methylation and demethylation in the promoter region, triggering gene transcription in the appropriate order in neural stem cells. Additionally, *HES* gene expression occurs in an oscillatory manner approximately every two hours [33,34] and also in the adult brain [35]. 

Thus, our research suggests that *HES1* may be involved in *PER2* gene expression, but the context of this co-regulation is unknown. It may be associated with neuronal and glial adaptation to environmental cues in the pre-optic area of the hypothalamus, especially when we follow the changes in position 2 (Figure 3) in each of the three examined groups. Very clear 12 h variation in the methylation level in the N group at night was twice as high in comparison to that in the day. The variable methylation disappeared in the control group adapted to constant darkness, and the levels for both examined times were the same. In the CO-treated group, the CO was added to infused plasma through the angular vein and restored clear 12 h variation in the methylation pattern in the examined position inside the N-box sequence. Although the difference was not two-fold as in the N group and the night level of methylation was only 1.5-fold when the day level was similar to the N group, the reason was the constant infusion of “chemical light” to the brain, which simulated a constant day. As a result, we observed a lower methylation level in the night than we had expected. Furthermore the discussed sequence (CACGCG) was followed by a sequence belonging to the recognized DNMT3a/MYC (CASSTG), which facilitated methylation/demethylation CpG positions. This situation was not observed in any other methylated position in this fragment. The oscillatory effect like the above concerning the CACGCG sequence was discovered in the N group in the next examined site (−2000–−2054 bp); the oscillatory effect disappeared in the C and CO-treated groups, and the methylation level increased to 80% in comparison to 60% in the N group (Figure 4). It seems that the farther the fragment was located from the site recognized by the DNMT3/TF, the smaller the dynamics of change were. 

The results of functional analyses (Figure 7) described the short and long promoter *PER2* gene sequence and showed data concerning the function of this sequence. In this experiment, we assumed that the number of (CANNTG) sequences would be crucial for the timing of the variable expression of this gene. Both the canonical E-box and D-box sequence were preserved because they are crucial for the autonomous oscillation of this gene [24,27]. According to our results, we found that the oscillatory rate depended on the number of E-boxes in the promoter sequence. Our data showed that the cycle of the longer promoter (22 E-boxes) was approximately six hours longer than that of the shorter promoter (11 E-boxes). It was visible when we compared the gene-expression pattern obtained from the qPCR analysis with the pattern of luciferase activity from the functional analysis (colored circles on the charts in Figure 7). This is consistent with the results described by Yamajuku et al. [24], and the sequences used for functional analyses were totally unmethylated despite being used as a template DNA with a natural methylation profile (from the pre-optic area of the hypothalamus of animals from the experimental group). The PCR reaction used to amplify this sequence could not restore the methylation profile, and then we obtained an unmethylated product when all E-box sequences and other structures presented in this fragment were opened for transcription factors recognizing these sites. We knew that each cell, even those used in in vitro analyses, had its own self-sustained biological clock, which oscillated despite a lack of central outputs from the main biological clock located in the SCN. When we used our *Per2:Luciferase* vector with the long and short promoter sequence, we assumed that the conserved internal clock elements would predominantly bind to E-boxes than other bHLH TFs. Indeed, we discovered that number of E-boxes influenced the rate of oscillation but not amplitude. Hence, the data show that methylation is one of the mechanisms that regulate the 12 h variable expression of the *PER2*. For example, annual changes in daylight over time are gradual (extended in time lengthening and shortening day time) so that de novo epigenetic regulation of the availability of E-boxes is possible. Moreover, carbon monoxide as a “chemical light” is involved in this regulation even without real light as photon waves. 

Finally, oscillations occurred in samples taken at the same time but that differed in the DNA template used (DNA template obtained in the middle of the day and the middle of the night). In natural conditions, this kind of variable is normal because it is a promoter of one of the biological clock genes with “programmed” methylation pattern. We showed that day vs. night changes in methylation occurred in this experiment. 

## 4. Materials and Methods

### 4.1. Experimental Animals

The study was conducted on twenty-two 8–10 month old gilts from March to April when the length of the night in natural conditions is close to the length of the day (LD12:12; natural lights-on 6 a.m.). Before the start of the experiment, animals in the control and experimental groups were adapted to complete constant darkness for 48 h.

1. Normal group (control of controls, *n* = 6)—animals were killed in the middle of subjective night and day, i.e., 24.00 (12 a.m.) (*n* = 3) and 12.00 (12 p.m.) (*n* = 3). Animals in this group were not subjected to any experiments and were kept under natural light conditions.
2. Control group (*n* = 8). Experiment was started about 12 p.m. Animals were kept in darkness for 48 h, and then a cannula was inserted into the angular eye through the dorsal nasal vein. They were kept for a further 72 h in total darkness, and plasma infusion was administered without the addition of CO. Infusion start was sequential: animals slaughtered about 12 p.m.—infusion start was 12 p.m., 72 h earlier, animals slaughtered about 12 a.m.—infusion start was 12 a.m., 72 h earlier. (See the description of the experiment.) After the infusion was completed, the animals were slaughtered as above (normal group).
3. Experimental group—also called CO-treated (*n* = 8)—Animals were kept in darkness for 48 h, and then a cannula was inserted into the angular vein of the eye through the dorsal nasal vein. They were kept in total darkness for a further 72 h, and plasma with the addition of CO was administered continuously. Infusion start was sequential: animals slaughtered about 12 p.m.—infusion start was 12 p.m., 72 h earlier, animals slaughtered about 12 a.m.—infusion start was 12 a.m., 72 h earlier. (See the description of the experiment.) After the infusion was completed, the animals were slaughtered as above (normal group).

### 4.2. Experiment Description

Days 1 and 2: Animals were adapted to constant darkness and also to a new environment (individual pens). Red light was used during feeding time, blood collection and infusion control.

Day 3: Animals was subjected to the surgical insertion of cannula into the jugular vein for blood collection and the dorsal nasal vein (vein which enters directly into ophthalmic sinus through the angular vein) for a 72 h infusion of blood plasma, either blank or with elevated CO.

Day 4: Recovery day after surgery, constant darkness.

Start of infusion.

Days 5 and 6: Infusion was continued for 72 h. Afterwards, slaughter occurred at 12 a.m. and 12 p.m. in constant darkness.

Days 7 and 8: After 72 h infusion in all experimental groups, animals were sacrificed through thiopental/KCl injection in the following sequences: *n* = 4 at 12 a.m.—starting around 11:50 a.m. and finishing the last at 1 p.m. (1 animal per 15 min); *n* = 4 at 12 p.m., starting around 11:50 p.m. and finishing the last at 1 a.m. (1 animal per 15 min).

Blood plasma was freshly prepared prior to infusion. For the elevation of CO in plasma, we used chromatographically clean CO in the amount of 0.8 cm^3^/50 mL plasma. CO was administered with a Hamilton’s syringe and a puncture-covered chromatographic vial when we had 50 mL blank plasma at room temperature. Afterwards, this plasma was mixed on a hematological roller. Infusion lasted 72 h in both groups at 8.3 mL/h. At this time, the animals were exposed to constant darkness. Infusion commenced on day 3 at 12 a.m. (*n* = 4) in. and 12 p.m. (*n* = 4) 

From our earlier experiments (data from PhD thesis by P. Gilun and publication by Koziorowski et al. 2012 [5]), we know that physiological CO level in LD 18:6 is 4.03 nmol/mL (maximum capacity of blood plasma for CO solubility is 13.54 nmol/mL), and in SD 6:18, it is 0.89 nmol/mL (the maximum capacity of blood plasma for CO solubility is 3.14 nmol/mL). We already know that the maximum capacity of CO solubility in blood plasma is only 3-fold greater than physiological level in both tested seasons. Thus, our experiments were safe and posed no risk of poisoning the CO-treated animals. All CO procedures were carried out in a ventilated chamber.

### 4.3. Surgical Procedures

**Figure 8:**
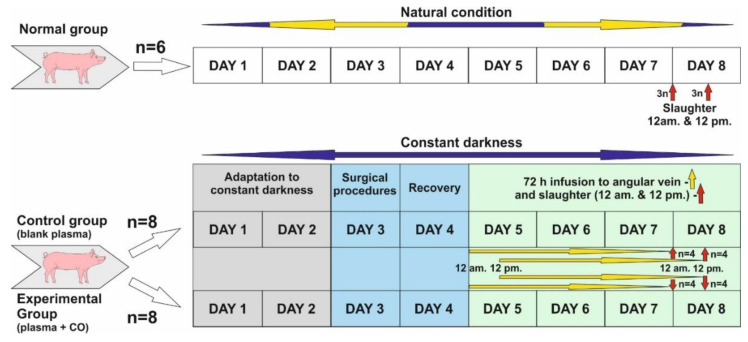
Schematic draw of experiment.

Cannula insertion to both the jugular vein and angular vein through the dorsal nasal vein was performed under general anesthesia: step 1—premedication with Stresnil (Janssen Pharmaceutica N.V., Beerse, Belgium) and ketamine (Biowet Puławy, Puławy, Poland) with atropine (Polfa, Warszawa, Poland) added i.m., step 2—general anesthesia with thiopental sodium (Sandoz GmbH, Kundl, Austria) added i.v. During and 24 h after surgery (recovery day), animals received fentanyl (Polfa, Warszawa, Poland) for pain relief. All procedures (Figure 8) were conducted in red light conditions, apart from the surgery itself when the animals’ eyes were covered with a black (light-proof) material.

### 4.4. Scheme of Experiment

#### 4.4.1. Tissue Sampling

After slaughter, the skull was opened, and the whole brain was prepared and dissected along the sagittal axis for the visualization of the hypothalamus. The hypothalamus was divided into the pre-optic, ventral and dorsal parts. For our research, we used the preoptic part of the hypothalamus, which contained suprachiasmatic nuclei (SCN). Tissues were collected at 12 a.m. and 12 p.m. and frozen in liquid nitrogen for further analysis.

#### 4.4.2. Nucleic Acids Isolation 

Frozen hypothalamic tissues was homogenized in a Fast-Prep 24 apparatus (MP Biomedicals, Illkirch-Graffenstaden, France) in Tri-Reagent (Merck, Kenilworth, NJ, USA). We obtained RNA and DNA from homogenized tissues using a protocol described by Chomczynski [36].

### 4.5. qPCR Analysis

For PCR analysis, 500 ng RNA (quality and amount measured by NanoDrop, ThermoFisher, Waltham, MA, USA) was reverse-transcribed (RT) using RevertAid H minus First Strand cDNA Synthesis Kit (ThermoFisher, Waltham, MA, USA) with random hexamers according to the manufacturer’s instructions. For the qPCR, we used *PER2* primers and a TaqMan probe designed with Primer Express software (Applied Biosystems, Waltham, MA, USA). The qPCR was conducted for forty cycles in 20 µL at Tm 60 °C at the default/predefined settings of an ABI 7900HT Real-Time PCR System (Applied Biosystems, Waltham, MA, USA) using Maxima Probe/ROX qPCR master mix (2×) (ThermoFisher, Waltham, MA, USA). Primers pairs are listed in Table S1.

### 4.6. Pyrosequencing Assay

In our experiments, we used a gene sequence (accession number NC_010457) from the Gene Bank and designed the pyrosequencing assays in four places (CpG rich) at the gene sequence of 2500 bp downstream from the start of translation (ATG). 

An amount (200 ng) of genomic DNA, extracted from the preoptic area of the hypothalamus, was bisulfite converted using an EZ DNA Methylation-Direct Kit (ZYMO Research, Irvine, CA, USA) according to the manufacturer’s protocol. Pyrosequencing assays for each selected promoter sequence were designed with PyroMark Assay Design v2.0 software (Qiagen, Germantown, MD, USA). The bisulfite DNA was PCR amplified using the PyroMark PCR Kit (Qiagen, Germantown, MD, USA). The pyrosequencing reaction was conducted on a PyroMark Q48 sequencer (Qiagen, Germantown, MD, USA). For pyrosequencing, we used a PyroMark Q48 AutoPrep Starter Kit (Qiagen, Germantown, MD, USA). Primers pairs are listed in Table S1.

### 4.7. Functional ANALYSIS

We designed the assays of functional analysis using a DNA sequence (Accession Number—NC_010457) from the Gene Bank and a bioinformatic tool (benchling.com). We used as a template DNA obtained from all experimental groups. According to the sequence analysis, we chose 2541 bp downstream ATG (start of translation), the optimal length of the sequence, as a long promoter version and 1290 bp downstream ATG as a short promoter version (Figure 6). Division of such a length was related to the content of the E-box (CANNTG), of which there were eleven in each section. For experiments we used psiCHECK2 vector with Renilla and Firefly luciferase, which we constructed using the Gibson Assembly method and NEBuilder bioinformatics tool (NEB.com). Primers for this method were synthetized in Eurofins Genomics (Germany), and the Gibson Assembly reaction was conducted with the HiFi DNA Assembly Cloning Kit (New England BioLabs, Ipswich, MA, USA) according to the manufacturer’s protocol. The obtained vector was transferred to electrocompetent *E. coli* bacteria and cultured on a plate with ampicillin medium. After colony growth, we checked the correctness of the Gibson Assembly reaction in the PCR colony. After selecting the proper colony, we transferred the bacteria to Luria Broth with ampicillin for the amplification of vectors, which, after eighteen hours, we isolated and cleaned with the use of the NucleoBond PC 500 Maxi kit for transfection-grade plasmid DNA (Macherey-Nagel, Düren, Germany), according to the manufacturer’s protocol. The obtained vectors were measured by NanoDrop and frozen for further analysis. Primers pairs are listed in Table S1.

### 4.8. In Vitro Analysis

The HEK293 cells were transferred with vector psiCHECK2 by lipofection (lipofectamin: ThermoFisher, Waltham, MA, USA). Afterwards, the cells were incubated for forty-eight hours in DMEM medium with additives (not synchronized with dexamethasone to allow natural circadian cell cycle activity), and harvesting started at 8 a.m. with six-hour intervals. The luciferase activity was measured by luminometer LumiStar Omega (BMG Labtech, Ortenberg, Germany) with Firefly and Renilla Luciferase Single Tube Assay Kit (Biotium, Fremont, CA, USA), according to the manufacturer’s protocol.

### 4.9. Statistical Analysis

Statistical analysis of the obtained results was calculated using one way ANOVA test followed by post hoc Tukey’s test and presented as the mean ± standard error of the mean (SEM) with statistical significance considered at *p* < 0.05 using GraphPad Prism 9 software (GraphPad, San Diego, CA, USA).

## 5. Conclusions

Twelve-hour variations in methylation levels were observed in natural conditions, disappeared when there was a lack of light and partially resumed as a result of a CO enriched infusion transferred to the hypothalamus via the humoral pathway. As well as changes in day vs. night sampling, the gene expression of the *PER2* gene was observed to be linked with methylation pattern changes. Hence, we suggest the CO may act as a chemical light signal and support the modulation of gene expression of *PER2* in an epigenetic way.

## 6. Scientific Potential

This paper showed the regulation of circadian expression of *PER2*, based on a proposed method of “programming” the DNA through its promoter methylation/demethylation. This kind of regulation explains the distinct expression of *PER2*, which is often observed in many experiments wherein the pattern of daily oscillation diverges from other Period and Cryptochromes genes. An example of such differences is a delayed increase in expression after glucocorticoid treatment compared to *PER1* gene [37]. The authors would like to specifically point out that CpG methylation was not considered a dynamic (circadian) gene-expression modulator and instead was merged with “learning” processes and with the fixation of pattern methylation of genes in response to the long-term impact of some cues. In conclusion, our study showed a method of daily modulation of *PER2* gene expression.

## Data Availability

All data are available from corresponding author.

## Supplementary Materials

The following are available online at https://www.mdpi.com/article/10.3390/ijms22157796/s1, Table S1. Sequences of primers used in experiment.
