# Supplementary material for: Role of Methylation in Period2 (PER2) Transcription in the Context of the Presence or Absence of Light Signals: Natural and Chemical—Studies on the Pig Model
Source: Int J Mol Sci. 2021 Jul 21;22(15):7796. doi: 10.3390/ijms22157796 (PMC8346033; doi:10.3390/ijms22157796)
Supplement: Supplementary file 1 [file ijms-22-07796-s001.zip › ijms-1246563-supplementary.pdf]

Table S1. Sequences of primers used in experiment

	Sequence of Primers and Probes	Gene Bank Accesion Number
**qPCR**		
Per2	Reverse – 5′ CATCTTCCACCGTCTCAAGCT 3′<br>Forward – 5′ AGCATCGGCCTCTAAGATGAAC 3′<br>Probe – 5′[6FAM] ACGTTAGCACGCACTTGACCTCGCTG [TAM]3′	**NC_010457**
Sdha	Reverse – 5′ CCAGTTGTCCTCCTCCATGTTC 3′<br>Forward – 5′ CTCTCTGAGGCCGGGTTTAA 3′<br>Probe – 5′[6FAM]TTACGAAGCTCTTTCCCACCAGATCACACA [TAM]3′	**XM_021076930**
**Pyrosequencing** (bisulfite converted DNA)		**NC_010457**
0–−79 bp	Reverse – 5′ TGGAAGGGATTATTTAGGAGGATA-3′<br>Forward – 5′ [BIO] TAACCCTCAAAACCCCCCACC-3′<br>Sequencing – 5′GTTTTTATTGAGGAAT-3′	
−1124–−1185 bp	Reverse – 5′ [BIO] ACCAATTCCCAAAACCAAATC 3′<br>Forward – 5′ GGGTGTGTTAAGGTGTGATTG 3′<br>Sequencing – 5′ ATTTATGTTTGATGAAGTTGT 3′	
−2000–−2054 bp	Reverse – 5′ [BIO] ATTTCTA ACAACCTATTTCCTCCAA 3′<br>Forward – 5′ AGAGTAAGTAGTGGTTAGTATAATGT 3′<br>Sequencing – 5′ GTTTTATATATAGTTGG 3′	
−2402–−2464 bp	Reverse – 5′ [BIO] CCCCTAAAACAAACCAAAACTAT 3′<br>Forward – 5′ GGATAGTATTTTTTAATAGTTGGGGT 3′<br>Sequencing – 5′ AATTTGAAAGTATAAATG 3′	
**Gibson Assembly**		
long version 2541 bp	Reverse – 5′ GCTTTTTGCAAAAGCCTAGGGCCCATCGCT 3′<br>Forward – 5′ AGAGGAACTTGGTTAGGTACGCTCAGAAAC 3′	
short version 1290 bp	Reverse – 5′ GCTTTTTGCAAAAGCCTAGGGCCCATCGCT 3′<br>Forward – 5′ AGAGGAACTTGGTTAGGTACCTGCCAGCCC 3′	
